# The Embedded Health Management Academic: A Boundary Spanning Role for Enabling Knowledge Translation: Comment on "CIHR Health System Impact Fellows: Reflections on ‘Driving Change’ Within the Health System"

**DOI:** 10.15171/ijhpm.2019.108

**Published:** 2019-11-11

**Authors:** Kathy Eljiz, David Greenfield, Robyn Taylor

**Affiliations:** Australian Institute of Health Service Management (AIHSM), University of Tasmania, Sydney, NSW, Australia

**Keywords:** Boundary Spanning, Integrated Knowledge Translation, Collaboration, Healthcare Organisational Improvement

## Abstract

Healthcare organisations are looking at strategies and activities to improve patient outcomes, beyond clinical interventions. Increasingly, health organisations are investing significant resources in leadership, management and team work training to optimise professional collaboration, shared decision-making and, by extension, high quality services. Embedded clinical academics are a norm in, and considered a strength of, healthcare organisations and universities. Their role contributes, formally and informally, to clinical teaching, knowledge sharing and research. An equivalent, but significantly less common role, addressing the management of healthcare organisations, is the embedded health management academic (EHMA). A stimulus encouraging this intertwined embedded academic role, in both clinical and managerial fields, is the demand for the translation of knowledge between academic and industry contexts. In this essay, we describe the EHMA role, its value, impact and potential for enabling healthcare organisation improvement. Focusing on the business of healthcare, the EHMA is a conduit between sectors, stakeholders and activities, enabling different organisations and experts to co-create, share and embed knowledge. The value and impact achieved is significant and ongoing, through the nurturing of an evidence-based management culture that promotes ongoing continuous improvement and research activities.

## Introduction

Healthcare organisations are increasingly looking at strategies and activities to improve patient outcomes, beyond clinical interventions. A key reason being that the global cost of healthcare is projected to rise from US$7.724 trillion to $10.059 over the 2017–2022 period.^1^ The push for the sustainability of healthcare organisations has led senior leadership teams to make the link between the effective functioning of their facilities and high quality, safe clinical care.^2,3^ This is recognition that focusing on improving the business of healthcare enhances the context, scope, and opportunities for the delivery of clinical care.^4^ Increasingly, therefore, healthcare organisations are investing significant resources in leadership, management and team work training to optimise professional collaboration, shared decision-making and, by extension, high quality services.^5,6^ Effective leadership and management training is grounded in understanding staff needs, both clinical and corporate, and tailoring development programs to match.^7^

To achieve health system improvement, we are witnessing a growth in the demand for collaboration and the translation of knowledge between sectors - industry and academia, stakeholders – policy-makers, clinicians and consumers, and activities – education and research.^8,9^ In the United Kingdom, for example, there has been a requirement for education and research to take on a more translational approach, eg, National Institute for Health Research Collaborations for Leadership in Applied Health Research and Care.^10,11^ Similarly, in China, there is growing attention being placed on multi-institution collaboration with the United States for increased clinical and translational research, eg, Joint Institute for Translational and Clinical Research.^12^ Whereas, in Australia research-education translation continues to expand via three mechanisms, including Centres of Research Excellence, Advanced Health Research and Translation Centres, and Clinical Networks.^13^ These three examples highlight Cassidy and colleagues^9^ recent point - that there is a pressing need to establish and maintain collaborative partnerships, allocate time for management research, and demonstrate the added value for both academic and healthcare organisations.

Healthcare needs conduits to make system improvements, connections and knowledge translation across sectors, stakeholders and activities.^14^ Embedded clinical academics are a norm in, and considered a strength of, healthcare organisations and universities. Their role contributes, formally and informally, to clinical teaching, knowledge sharing and research.^15^ Clinical academics are ‘boundary spanners’^10^ who bridge the healthcare service delivery and education sectors. These roles have also been termed ‘researcher in residence’^16^ or an ‘embedded researcher,’^17^ as they provide a direct link between the service delivery frontline to education organisations. When applied effectively, embedded research has the capacity to make informed changes to practice,^17^ thereby improving the quality of care delivered. An equivalent, but significantly less common role, addressing the management of healthcare organisations, is the embedded health management academic (EHMA). This role undertakes teaching, knowledge sharing and research activities focused on improving the organisation and management of safe services and facilities. A stimulus encouraging this intertwined embedded academic role, in both clinical and managerial fields, is the demand for the translation of knowledge between academic and industry contexts in developed countries.^10-12,18^ Whilst relatively new, the need for the boundary spanning role is emerging across different countries, helping to overcome barriers between the health service industry and academic institutions.

One country where the embedded researcher is being trialed is in Canada, through the Canadian Institutes of Health Research Heath System Impact Fellowship scheme. That is, positions that are co-located between healthcare organisations and universities to foster health system transformation.^19^ The scheme, having been operating for two years with 95 Fellows placed, is reported as being successful in meeting organisational needs and providing high quality post-doctoral training.^20^ However, aligning university and organisational outcome metrics together continues to pose difficulties.^21^ Nevertheless, there is recognition of the need to create environments and organisations that can “occupy the middle ground, interpreting and translating messages that should flow both ways between researchers and policy-makers and practitioners.”^22^ In this space, three integrated knowledge translation components are promoted through partnership relationship management, allocating time for applied research, and outlining the value for both university and health organisations.^9^

Knowing embedded researchers are needed and being able to create collaborations to allow them to succeed is not easily reconciled. In this paper, we describe how a “middle ground” and embedded research role was created, nurtured and succeeds. That is, the EHMA role, its value, impact and capacity for enabling healthcare organisation improvement and integrated knowledge translation. The development and the enactment of the EHMA role, within a healthcare-academic collaboration, which is the ‘Australian Institute of Health Service Management’ (AIHSM), is explained. This includes addressing the benefits and challenges for the university and healthcare organisational partners.

## The Embedded Health Management Academic Role

The AIHSM is formed through the collaboration of the University of Tasmania, Tasmanian School of Business and Economics and various healthcare organisations. In doing so, the collaboration integrates expertise and skills of healthcare academics and executives. The Institute’s purpose is to develop healthcare employees organisational and managerial knowledge and skill; this includes, enhancing their abilities in systems and critical thinking, leadership, information retrieval and assessment, and problem-solving strategies.

To advance healthcare system improvement, the creation, sharing and translation of knowledge is key.^19,23^ Focusing on business models of care can release opportunities for innovation. The Institute focuses on the functional level, that is, the enhancement of leadership and management capabilities across an organisation. The Institute seeks to achieve enhancements to the business of healthcare organisations through knowledge symmetry between sectors, stakeholders and activities.

There is opportunity for the AIHSM and EHMA to develop further as recent Australian Government policy is actively promoting universities and industry to work more closer together. There are funding incentives for university-industry engagement through two grant mechanisms: university research block grants,^24^ for academics; and, translational research grants,^25^ for health professionals. These provide a direction for greater engagement and collaboration into the future.

The EHMA role has been developed, and operates, within the AIHSM context. The EHMA name reflects the focus, characteristics and purpose of the role: health sector organisation and management; embedded, co-funded and co-located within a health organisation; and, directed at delivering academic education and research activities. The EHMA role traverses organisational and professional boundaries facilitating engagement with staff regardless of discipline, position, or service location. The EHMA role facilitates the networking of staff, thereby breaking down silos across formal and informal structures and service locations.^26^ In this way, the EHMA role contrasts with the primary role of the clinical academic, which focuses on developing defined clinical capabilities, that is, the skills and knowledge of medical, nursing or allied health professionals.

The EHMA role is enacted through four integrated objectives (Table 1): promoting engagement and networks; stimulating knowledge co-development and translation; encouraging enhancements and improvements; and, furthering distributed leadership and emotional intelligence. The specific EHMA activities at any time are determined by the needs of the health organisation and capabilities of the specific executives, managers and academics collaborating. It is an unfolding flexible arrangement continually renegotiated over time as both develop and change.

**Table 1:** Key EHMA Objectives and Activities

**Objective**	**Activity**
**Engagement and networks** Collaboration to realise opportunities across the health system	Networking between academic and industry experts and frontline staff
	Professional collaboration at intra-organisational and inter-organisational levels
**Knowledge co-development and translation** Reciprocal relationship based on exchange of expertise	Maturing and promoting an ongoing learning culture
	Developing, facilitating and administering Masters level coursework with industry partners
	Developing and facilitating management, leadership and research sessions
	Co-construction of literature reviews and presentations
	Reciprocal relationship of resourcing, including in-kind support, access to materials and expertise exchange
	Industry and academic abstracts, presentations and publications
**Enhancements and improvements** Aligning the organisation and management of care for improved patient outcomes	Translational research and evaluation projects aligned with organisational strategic plans
	Development of a management research culture
	Program evaluations including design, analysis and reporting
**Distributed leadership and emotional intelligence** Facilitating critical thinking skills to self-manage, lead and identify opportunities for improvement	Mentoring and support
	Enhancing capacity in systems and critical thinking
	Professional development and talent management identification
	Development of individuals self-awareness and self-regulation skills

Abbreviation: EHMA, embedded health management academic.

Performing the EHMA role effectively requires an individual with a flexible, open and collaborative mindset, and behaviours (Table 2). The reason being that the EHMA role works in complex healthcare organisations, often in environment of uncertainty, across organisational and professional boundaries. The EHMA works with health professionals to develop their critical and systems thinking, undertake research-quality improvement projects, with a knowledge translation goal, and enhance the emotional intelligence of individuals.

**Table 2:** EHMA Mindset and Behavioural Characteristics

**Domain**	**Attributes**
Mindset	Approaching tasks in a flexible and creative manner
	Ability to deal with complexity
	Willingness to deal with ambiguity
	Attentiveness to the changing context
Behaviours	Acting in a collaborative manner
	Demonstrating team work
	Performing boundary spanning activities
	Developing and nurturing networks
	Active listening with colleagues
	Asking questions and exploring ideas, options, and possibilities

Abbreviation: EHMA, embedded health management academic.

The EHMA role has an insider-outsider^27^ position in a health organisation with the task of being a knowledge spanner. By contributing to both health services and university institutions knowledge needs, the EHMA role co-creates, mobilises and diffuses knowledge.^19,28^ The outcomes are achieved through a long-term collaborative partnership delivering postgraduate education and research, and organisational development activities. One critical activity that the EHMA facilitates is the translational research project, which is a capstone of the AIHSM Masters programs. Using a research lens to address organisational strategic priorities, healthcare employees work with academic and industry advisors to make tangible recommendations for improving organisational performance. Quality improvement initiatives are strengthened through the grounding in and integration with peer reviewed and organisational data. The co-development of evidence informed recommendations is how healthcare organisations can make enhancements, thereby achieving the goal of knowledge translation.

## Outcomes for Healthcare and University Organisations

The collaboration between healthcare organisations and the university is the formation of the AIHSM and the EHMA role, and positive outcomes for each (Table 3). The outcomes for health organisations are at the individual level, including students and mentors/executives, and structural-systems level, including teams, services, and networks. Working within an organisation the EHMA progresses the development of an evidenced based management culture through the collaboration with executives and managers and focusing upon their strategic priorities. The EHMA endeavours to nurture a matrix of relationships across services and locations to promote an organisational management improvement research culture. The outcomes for the university are at the individual academic, AIHSM team and program levels. Continual, and mutual, knowledge development and embedding are achieved, for all levels, through the collaboration. In this way, the AIHSM and the EHMA role is fulfilling the call by Cassidy and colleagues^9^ for integrated knowledge translation through collaborative relationships.

**Table 3:** Outcomes for Healthcare and University Organisations

**Organisation**	**Component**	**Outcomes**
Healthcare	Students	• Postgraduate management education leading to a university qualification • Research training promoting outputs including academic and industry presentations, reports and papers • Development of critical thinking and problem-solving skills^6^ • Exposure to senior executives and managers, industry experts resulting in creation of networks within and across organisations
	Mentors and executives	• Sharing of knowledge through an education role • Environment that encourages and challenges their thinking, behaviours and practice through the application of reflexivity^29^ • Contribute to academic coursework and research studies
	Succession planning	• Talent management identification of staff who are performing at work and in the postgraduate programs • Identification and nurturing of deep smart individuals^30^ • Influence strategy for succession planning across the organisation
	Networks	• Shared matrix learning environment between corporate and clinical staff and junior-senior staff helping to break down silos for improvements in the delivery of patient care outcomes^26^ • Development of a cohort of professional/expert managers and executives
	Culture	• Nurture a management culture that encourages ongoing learning through training, assessments and research projects related to strategic vision • Increased knowledge translation of evidence-based management
University	Individual	• Ongoing updating of industry challenges and needs • Alignment of research and teaching interests with industry priorities • Environment to test and refine research ideas and teaching concepts via immediate feedback
	Team	• Collaborative, supportive work culture • Co-development of the knowledge spanner^14^ EHMA role • Foster and apply the co-creation of knowledge between academia and industry^31^
	Program	• Promote engagement between industry and academia across and between organisations • Develop academic programs and training targeting industry priorities

Abbreviation: EHMA, embedded health management academic.

These outcomes present benefits and challenges for both healthcare and university organisations. The benefits of the intertwined role are numerous, including: co-construction of knowledge and close engagement between industry and academia^32^; impactful research for the end-user^33^; and, an allocation of resources based on evidence.^16^ Conversely, the challenges for the AIHSM and EHMA include: time constraints; securing and retaining funding^21^; managing different, competing demands^21,34^; appropriate performance measures^35^; and, identifying potential staff with motivation, mindset and capacity to take on the role. The barriers, enablers and challenges related to the EMHA role are very likely to reflect those of other similar roles, such as clinical academics and embedded researchers.

## Conclusion

Healthcare-academic collaborations with roles, such as the AIHSM with the EHMA, can flexibly enable different organisations and experts to co-create, share and embed knowledge, in all participants. The AIHSM is a practical example of the “middle ground” needed to achieve integrated knowledge translation: a collaboration that unites academics, practitioners and policy-makers. The EHMA contributes to the development of healthcare employees organisational and managerial knowledge and skills. Working with staff the EHMA facilitates professional development focused on enhancing individual’s abilities in systems and critical thinking, leadership, information retrieval and assessment, and problem-solving strategies.

Focusing on the business of healthcare, the EHMA is a conduit between sectors, stakeholders and activities for the improvement of services and facilities. The value and impact achieved is significant and ongoing, through the nurturing of an evidence-based management culture that promotes ongoing continuous improvement and research activities. Future research is needed into the barriers, enablers and contextual conditions that enhance the efficacy of the EHMA role to promote the efficient delivery of safe, high quality care.

## Acknowledgements

We wish to acknowledge and thank the partner organisations of the Australian Institute of Health Service Management (AIHSM), University of Tasmania, Sydney, Australia for their ongoing support.

## Ethical issues

Not applicable.

## Competing interests

Authors declare that they have no competing interests.

## Authors’ contributions

All three authors contributed to the intellectual development of ideas, sourcing literature, writing the manuscript and editing the manuscript.
